# Treatment of Poultry Slaughterhouse Wastewater (PSW) Using a Pretreatment Stage, an Expanded Granular Sludge Bed Reactor (EGSB), and a Membrane Bioreactor (MBR)

**DOI:** 10.3390/membranes11050345

**Published:** 2021-05-08

**Authors:** Honeil Basile Meyo, Mahomet Njoya, Moses Basitere, Seteno Karabo Obed Ntwampe, Ephraim Kaskote

**Affiliations:** 1 Bioresource Engineering Research Group (BioERG), Department of Chemical Engineering, Cape Peninsula University of Technology, P.O. Box 1906, Bellville 7535, South Africa; oneil_meyo@hotmail.com (H.B.M.); mahomet.njoya@gmail.com (M.N.); 2 Academic Support Programme for Engineering in Cape Town (ASPECT) & Water Research Group, Department of Civil Engineering, University of Cape Town, Private Bag X3, Rondebosch 7701, South Africa; 3 Center of Excellence in Carbon-Based Fuels, School of Chemical and Minerals Engineering, North-West University, Private Bag X1290, Potchefstroom 2520, South Africa; karabo.ntwampe@uct.ac.za; 4 Malutsa (Pty) Ltd., c/o Oude Pont and Meent Street (Malutsa House), Wellington Industrial Park, Wellington 7655, South Africa; ephraimk@malutsa.co.za

**Keywords:** chemical oxygen demand (COD), expanded granular sludge bed reactor (EGSB), fats, oil, and grease (FOG), membrane bioreactor (MBR), poultry slaughterhouse wastewater (PSW), total suspended solids (TSS)

## Abstract

This study presents the biological treatment of poultry slaughterhouse wastewater (PSW) using a combination of a biological pretreatment stage, an expanded granular sludge bed reactor (EGSB), and a membrane bioreactor (MBR) to treat PSW. This PSW treatment was geared toward reducing the concentration of contaminants present in the PSW to meet the City of Cape Town (CoCT) discharge standards and evaluate an alternative means of treating medium- to high-strength wastewater at low cost. The EGSB used in this study was operated under mesophilic conditions and at an organic loading rate (OLR) of 69 to 456 mg COD/L·h. The pretreatment stage of this laboratory-scale (lab-scale) plant played an important role in the pretreatment of the PSW, with removal percentages varying between 20% and 50% for total suspended solids (TSS), 20% and 70% for chemical oxygen demand (COD), and 50% and 83% for fats, oil, and grease (FOG). The EGSB further reduced the concentration of these contaminants to between 25% and 90% for TSS, 20% and 80% for COD, and 20% and >95% for FOG. The last stage of this process, i.e., the membrane bioreactor (MBR), contributed to a further decrease in the concentration of these contaminants with a peak removal performance of >95% for TSS and COD and 80% for the FOG. Overall, the system (pretreatment–EGSB–MBR) exceeded 97% for TSS and COD removal and 97.5% for FOG removal. These results culminated in a product (treated wastewater) meeting the discharge standards.

## 1. Introduction

The contamination of clean water sources contributes to the global water crisis. Therefore, the treatment and reuse of wastewater is indispensable. Additionally, adequate management of water sources is critical in semiarid and dry regions of countries such as South Africa (SA) [1,2]. To protect both amphibian and earthbound living animals, the wastewater needs to be treated effectively before discharging it into freshwater sources [3,4]. The quality of wastewater generated from various industrial facilities depends on prevailing operations in those industries and the quantity of contaminants produced during these operations [5]. Depending on the characteristics of the given industrial wastewater, various methods can be used for its treatment. Therefore, biological treatment is deemed the most suitable for wastewater laden with high organic matter, suspended solids, fats, oil, and grease (FOG), macronutrients, and pathogens [6,7]. In this regard, the treatment of poultry slaughterhouse wastewater (PSW), which is the primary focus of this study, can, therefore, be efficiently treated using a biological system [8,9]. Pollutants in the PSW, if discharged untreated to the environment, can cause eutrophication and deoxygenation of receiving water bodies, which can harm the health of humans, animals, and plants. Therefore, it is important to treat such wastewater before it is discharged into the environment [3,10].

SA is currently experiencing challenges associated with water shortages, which the poultry industry (producing 1.93 million metric tons in chicken meat in SA for the year 2020) could solve by developing advanced treatment processes to treat the wastewater produced to meet national legislation and municipal discharge standards, including local government regulations. These regulations are implemented such that sustainable wastewater treatment technologies are developed and used to lessen potable water usage while protecting the environment. This motivated several industries to devise new methods of water reclamation to lessen reliance on currently available water resources [11,12].

Several recent studies investigated the treatment of PSW using biological systems. One of these studies encountered challenges when an expanded granular sludge bed reactor (EGSB) combined with anoxic–aerobic tanks was used, whereby the system experienced sludge washout when the influent had high FOG and a high suspended loading rate (SLR) [11]. Furthermore, a similar study was done by Sheldon and Erdogan [13], who reported that an EGSB coupled with a membrane bioreactor (MBR) achieved excellent results for treating soft-drink industry wastewater, removing most of the contaminants, including macronutrients, when the EGSB was used as a primary biological treatment unit. This culminated in this research study, which focuses on treating PSW using a pretreatment stage–EGSB–MBR system. Similarly, Zhang et al. [14] achieved a 91% total chemical oxygen demand (tCOD) removal rate at a hydraulic retention time (HRT) of 48 h and an organic loading rate (OLR) of 17.5 kg COD/m^3^·day at an average operating temperature of 35 °C using an EGSB, although the system was designed for treating palm-oil mill effluent. This system was not coupled with an MBR.

Due to financial constraints and a lack of promotion of advanced and affordable wastewater treatment options in SA, particularly for PSW, there is an urgent need to develop effective and low-cost solutions for high-strength wastewater treatment, particularly PSW. This study was aimed at investigating the effectiveness and performance of using a miniaturized lab-scale plant consisting of a pretreatment stage and an EGSB coupled with a MBR, for wastewater treatment by poultry slaughterhouses globally.

## 2. Materials and Methods

### 2.1. Poultry Slaughterhouse Wastewater (PSW) Sampling

The PSW used in this study was collected from a poultry slaughterhouse situated in the Western Cape province of SA. The poultry slaughterhouse processes a large quantity of birds, which in turn generates a large quantity of PSW [15]. The PSW generated comes from numerous processes (killing, bleeding, scalding, defeathering, etc.) and is partly treated onsite to meet the City of Cape Town (CoCT) discharge standards [16]. The PSW collected was sampled during peak production using 25 L polypropylene containers and stored in a refrigerator at 5 °C to minimize acidification. The sampling of the PSW was done 3 days a week and used as a feed to the miniaturized lab-scale plant designed.

### 2.2. Ecoflush^TM^—A Supplementation Agent for the Pretreatment Stage

Ecoflush^TM^ is a commercial product that is supplied in SA by Mavu biotechnologies (Pty) Ltd. as an assemblage of consortia producing hydrolases. The microorganisms were isolated from soil and subsequently grown and stored in a physiologically dormant state. When exposed to a rich organic source, such as PSW, they are resuscitated to produce enzymes primarily for FOG hydrolysis. The product also contains glaucids and fundamental amino acids that invigorate the natural tendency of specific microorganisms to produce enzymes associated with the hydrolysis of hydrocarbon constituents constituting the organic matter. Ecoflush^TM^ also oxidizes NH_3_ into NO_3_^−^ and NO_2_^−^. It also eliminates NH_3_, including odor-producing organisms, while rapidly diminishing the population of pathogenic microbes [17]. Ecoflush^TM^ weakens the hydrocarbon chains in FOG and complements other organisms that are prevalent in high-strength wastewater while reducing H_2_S-producing microorganisms, thereby rapidly decreasing odor [17].

### 2.3. Operation of the Pretreatment–EGSB–MBR System

**Figure 1:**
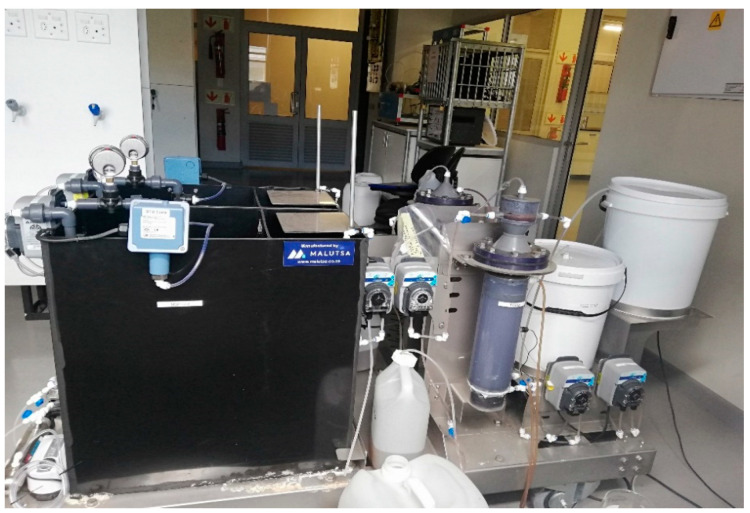
Poultry slaughterhouse wastewater (PSW) miniaturized lab-scale plant setup.

The PSW treatment system consisted of a biological pretreatment stage whereby raw PSW was mixed with Ecoflush^TM^ for biodelipidation before the PSW entered a holding feed tank used to supply the PSW to the EGSB as a primary organic matter removal system. The two stages were coupled with an MBR as the final treatment stage for a reduction in residual organic matter and total suspended solids (see Figure 1).

### 2.4. Pretreatment Tank Preparation

A mixture of the Ecoflush^TM^ (20 mL) and ~20 L of raw PSW (0.1% *v*/*v*) was used in the pretreatment tank for a reduction in FOG through biodelipidation and to induce bioflocculation of suspended particles. For the activation of the microbial community in the Ecoflush^TM^-supplemented pretreatment tank, the mixture was aerated by air stone spargers for 24 h at room temperature. After aeration, the air sparging was stopped such that the aggregated FOG and suspended solids were flocculated, before the PSW entered the feed tank (holding tank) of the EGSB. The PSW in the holding tank was analyzed for dissolved oxygen (DO), FOG, potential of hydrogen (pH), COD, electrical conductivity (EC), total dissolved solids (TDS), and total suspended solids (TSS). The PSW was thereafter fed to the EGSB.

### 2.5. EGSB Reactor System Used

**Figure 2:**
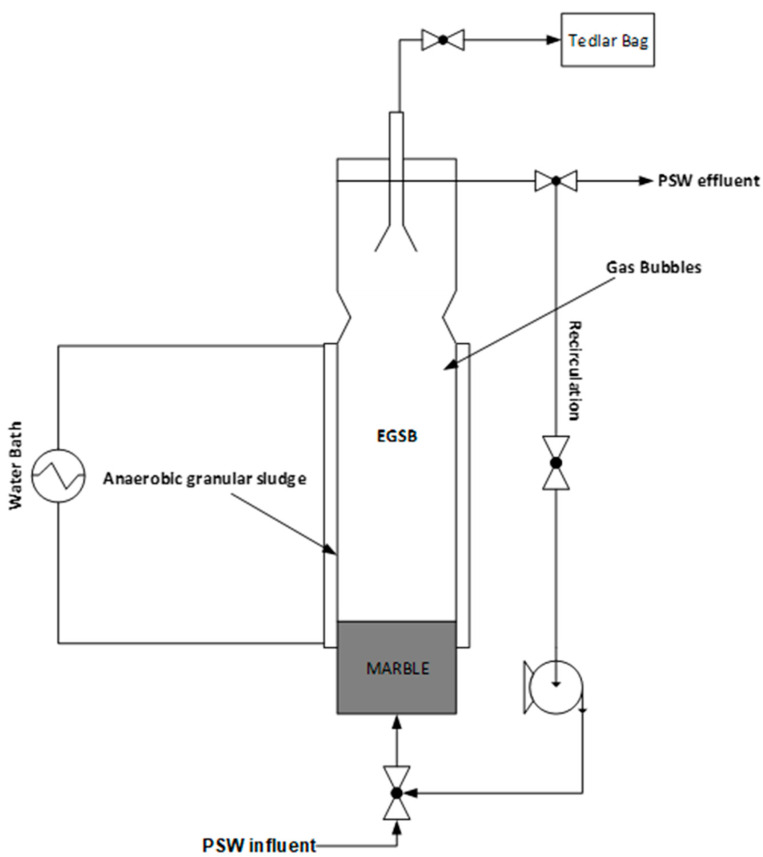
Schematic representation of the EGSB used.

The EGSB material of construction was a clear cylindrical polyvinyl chloride (PVC) column with a tapered bottom and a working volume of 2 L. The height was 0.6119 m with an internal diameter of 0.11 m. Ceramic marbles (0.0814 m) were used as packing material for the underdrain of the EGSB for sludge retainment. The recycle on the EGSB was utilized to regulate the PSW up-flow velocity of 0.1 m/h and bed expansion inside the EGSB, to prevent clogging of the underdrain in the bioreactor, and to better mix both the PSW and the sludge [18]. The EGSB was fed with PSW from the bottom using the Antech aspendose A 5.1L/0.5B peristaltic pump purchased from Enelsa in Turkey, Antalya. The product coming from the EGSB was sampled using 2 L polypropylene bottles subsequent to analyses. The EGSB was operated at a range of 35–37 °C, with the temperature being maintained using a heating jacket connected to a water bath maintained at 37 °C. To reduce heat loss to the environment, the EGSB was insulated (see Figure 2).

### 2.6. Inoculation of the EGSB

The inoculation of the EGSB was done by first putting the underdrain, followed by the addition of 0.4 L of activated sludge that was sampled from an anaerobic reactor in operation at the South African Breweries (Newlands, South Africa); thereafter, 1.6 L of raw PSW was added to the EGSB. The PSW, which was kept in a fridge at 5 °C, was incubated at 37 °C prior to use. Thereafter, six cups of Nestle Lactogen starter infant formula powdered milk were added to 400 mL of sterile distilled water to prepare a milk solution, with 200 mL of the milk solution being added as an organic source to sustain the sludge microbes for rapid growth [19].

### 2.7. Operating Conditions of the EGSB

The EGSB was kept at 35–37 °C during the 77 days of operation. The pretreated PSW in the holding tank was fed to the EGSB after 72 h of inoculation (stagnation period without PSW supplementation) to allow the volatile organic compounds (VOCs) to dissipate from the bioreactor mixture, as well as for DO reduction before the PSW was fed to the reactor [20]. The EGSB was run using a batch-fed strategy of PSW supply for 4 h/day for 7 days for microbial acclimatization, in order for the microbes to familiarize themselves with the PSW. This was done to achieve microbial growth, as the microbes in the EGSB needed nutrients for them to grow; after that acclimatization period, the bioreactor was run continuously throughout the study. The EGSB feed flow rate was 0.35 L/h with a hydraulic retention time (HRT) of 5.71 h, which was kept constant throughout the study.

### 2.8. Membrane Bioreactor (MBR) Used

**Figure 3:**
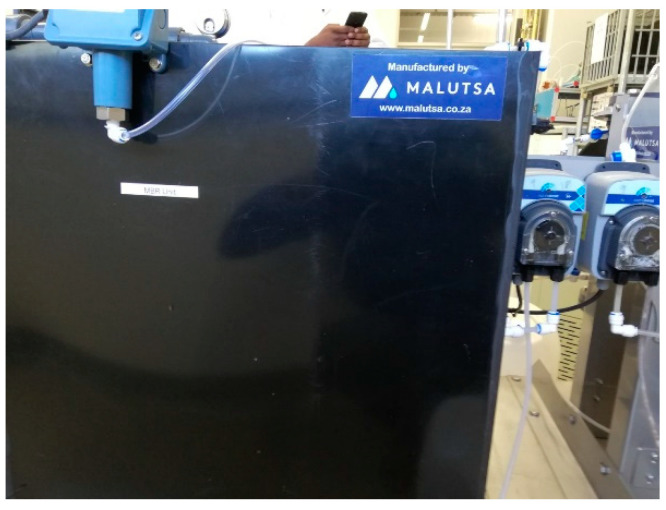
The MBR unit used.

**Figure 4:**
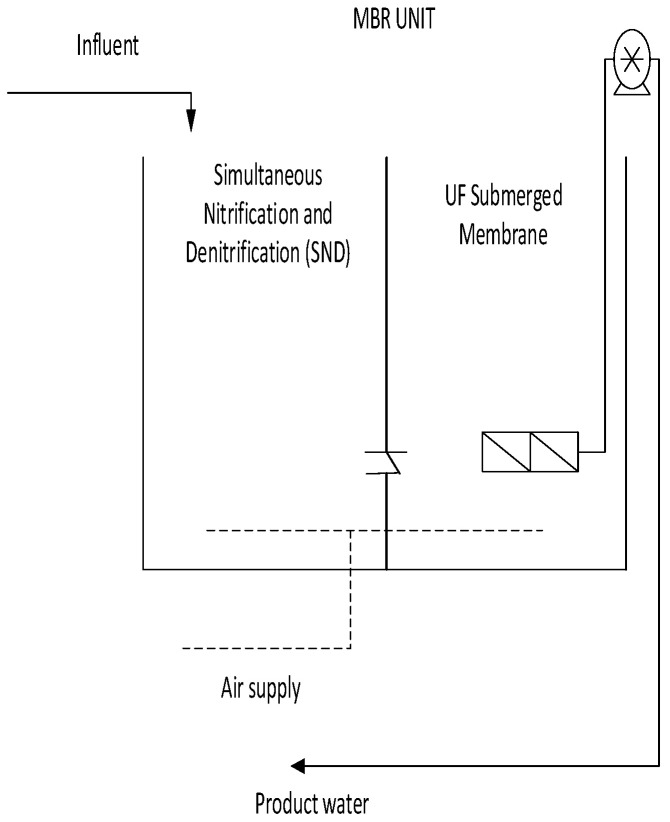
Schematic representation of the MBR unit used.

The MBR unit used had a rectangular container (working volume of 120.51 L) with embedded membranes within it. For the MBR, NADIR^®^ UP150 membranes were used. The membranes were composed of a hydrophilic polyether-sulfone (PES) sheet with a nominal pore size of ~0.04 µm, operated in a dead-end filtration mode (see Figure 3 and Figure 4). Inside the MBR unit, there was a mesh to cover the membranes to avoid clogging of the MBR unit by washout material from the EGSB. Sodium metabisulfite (SMBS) was used to preserve the membranes to avoiding microbial growth. The HRT was controlled by the Antech aspendose A 5.1L/0.5B peristaltic pump purchased from Enelsa in Turkey, Antalya. For aeration, a Regent^®^ RE-9500 air pump (Dolphin pumps, Cairo, GA, USA) was used to supply air into the MBR unit. A simultaneous nitrification and aerobic nitrification (SaND) compartment, as reported by Rinquest et al. [4], was incorporated within the setup.

### 2.9. Inoculation and Operating Conditions of the Membrane Bioreactor (MBR)

**Figure 5:**
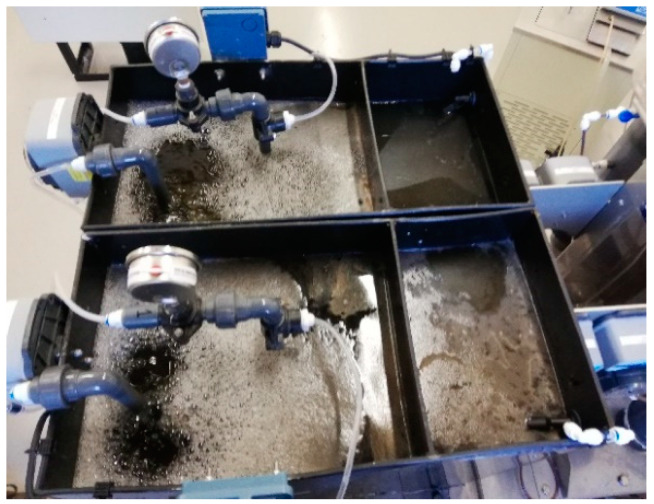
The MBR unit with a membrane compartment and an SNaD during inoculation.

The inoculation of the MBR unit was done by introducing 90 mL of the Ecoflush^TM^, followed by 90 L of water and 10 L of raw PSW (see Figure 5). The acclimatization period took 3 days; then, the EGSB effluent was introduced as feed into the MBR unit. Parameters such as temperature, pH, TDS, conductivity, and DO were measured within the MBR.

### 2.10. Sample Collection and Analyses for the Lab-Scale Plant

Throughout the study, a volume of either treated or untreated (to be fed to another unit) wastewater was analyzed for temperature, pH, COD, biological oxygen demand (BOD), electric conductivity, alkalinity (CaCO_3_), fats, oil, and grease (FOG), total dissolved solids (TDS), total suspended solids (TSS), dissolved oxygen (DO), and volatile fatty acids (VFAs) [21,22].

### 2.11. Analytical Methods for the Lab-Scale Plant Samples

All samples were analyzed for characteristic parameters at the CoCT scientific services laboratory, according to the standardized American Public Health Association (APHA) methods [21].

## 3. Results and Discussion

### 3.1. Pretreatment Stage Performance

**Figure 6:**
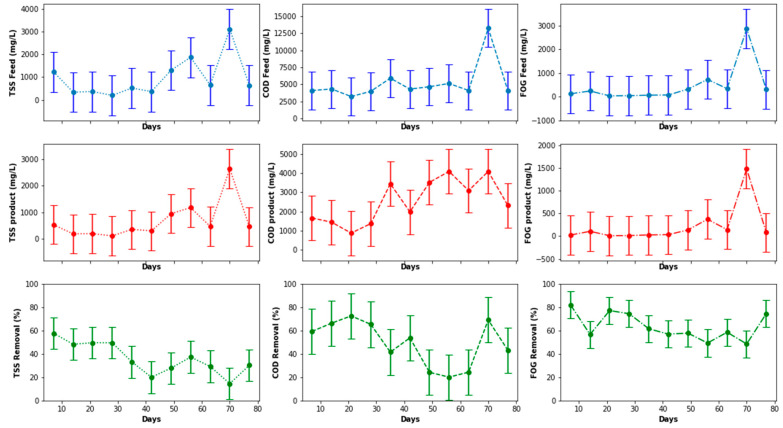
Pretreatment stage performance determined using COD, TSS, and FOG removal.

**Figure 7:**
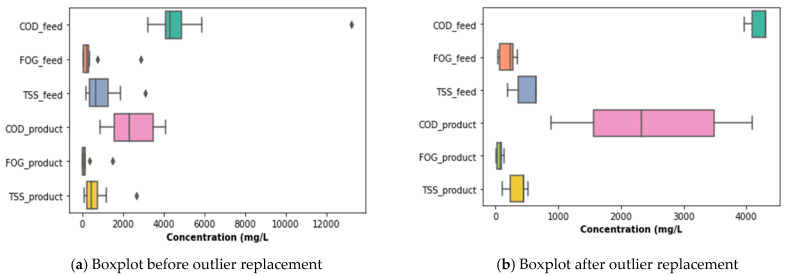
Boxplots of the distributions of TSS, FOD, and COD before and after outlier replacement.

Figure 6 presents the PSW quality characteristics from the pretreatment stage, accounting for fluctuations in the percentage removal determined from concentrations of TSS, FOG, and COD at the inlet and outlet. It was observed that the quality of the PSW feed and product fluctuated considerably, with a noticeably high concentration of the COD, TSS, and FOG on the 70th day of operation. This was attributed to various factors, including a significant change in the quality of the PSW fed to the pretreatment stage. This change could be directly related to the prevailing activity in the poultry slaughterhouse at the time of the sample collection, as a result of which the PSW may have contained more organic matter than normal. To assess the distribution of the sampling points with respect to the COD, FOG, and TSS, a boxplot was plotted, as illustrated in Figure 7, from which outliers can be noticed for each of the parameters for both the feed and the product of the PSW pretreatment stage. To correct the distribution of these data points and remove the noise from the data, various data processing techniques can be used such as the evaluation of the *Z*-score, the use of a standard scaler, or the application of the interquartile rule to identify and replace/delete outliers. The interquartile rule was selected for this study. Furthermore, the outliers were replaced instead of being deleted due to the size of the dataset. The outlier identification and replacement using the interquartile rule resulted in the distribution provided in Figure 7a,b, from which a distribution was smoothed with the replacement of the outliers with the median value of each parameter evaluated.

**Figure 8:**
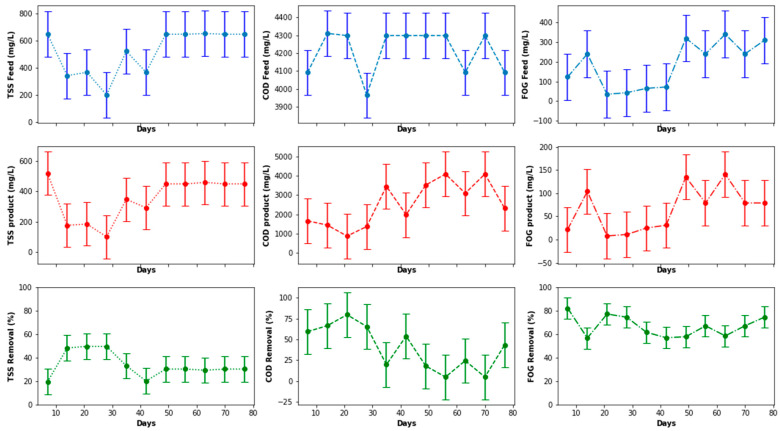
Pretreatment stage performance assessed using COD, TSS, and FOG concentration removal.

As illustrated in Figure 8, the peaks noticed for the inlet FOG, TSS, and COD in Figure 6 were eliminated and, thus, contributed to a reduction in the data distribution range for better analysis in order to elucidate a clear representation of the features of the PSW pretreatment. As observed in Figure 8, the pretreatment stage had an FOG removal of 55% to 85%. In addition to the pretreatment tank, the employment of star screens can contribute to the removal of a significant quantity of floating fats contained in the PSW. Furthermore, the pretreatment stage contributed significantly COD and TSS reduction, whereby the percentage removal oscillated between 20% and 50% for TSS and 10% and 80% for the COD. A further reduction in these wastewater quality characteristics can be improved with a further treatment process, i.e., biological treatment, thus motivating the use of the EGSB and the MBR.

### 3.2. Expanded Granular Sludge Bed Reactor (EGSB) Performance

**Figure 9:**
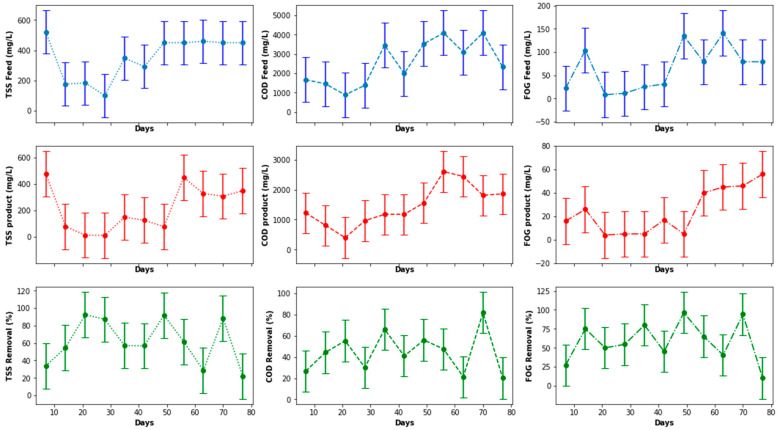
EGSB performance with respect to COD, TSS, and FOG removal.

**Figure 10:**
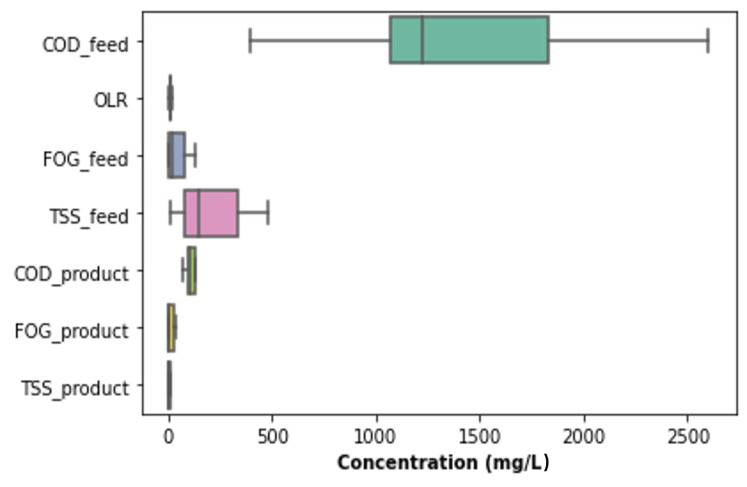
Boxplot of the EGSB parameters.

Before evaluating the performance of the EGSB in terms of COD, TSS, and FOG removal, as displayed in Figure 9, a boxplot of these values was plotted to visually detect possible outliers. The interest was specifically in the product stream generated from the EGSB, because the product would have a greater influence of the MBR performance. As illustrated in Figure 10, there were no outliers for the parameters quantified, including values observed for the organic loading rate (OLR). Therefore, no data processing or adjustment was required, as observed in Figure 9, with insignificant variations in the concentrations of the COD, TSS, and FOG in both the inlet and the outlet of the EGSB, which was attributed to the stability and performance of the pretreatment unit including the anaerobic bacteria within the anaerobic granular bed. Such a performance can be influenced by the competition between sulfate-reducing bacteria and methane-producing bacteria, including the accumulation of inhibitors within the anaerobic granular bed or other environmental factors that can prevent the anaerobic sludge granules to grow to maturity. Overall, with a consistent feed, it is possible to control most of these parameters during the anaerobic digestion stage. However, the temperature and the pH inside the bioreactor were continuously monitored and remained within the mesophilic range in terms of the temperature, while the pH fluctuated in the range 6.5 to 8. The expected performance trend for such a system would steadily increase over time, particularly for the removal of COD, TSS, and FOG.

**Figure 11:**
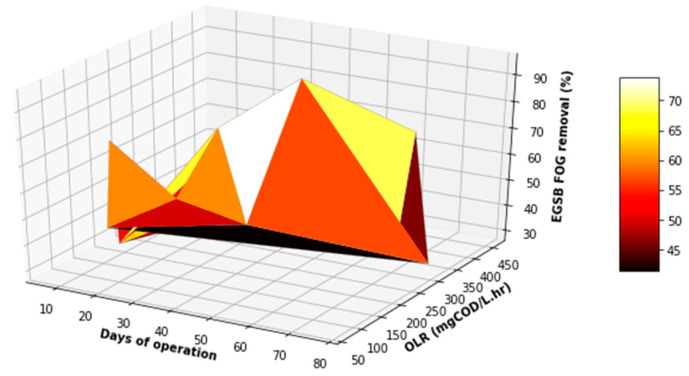
Variation in FOG removal with differentiated OLR during the EGSB operational time.

**Figure 12:**
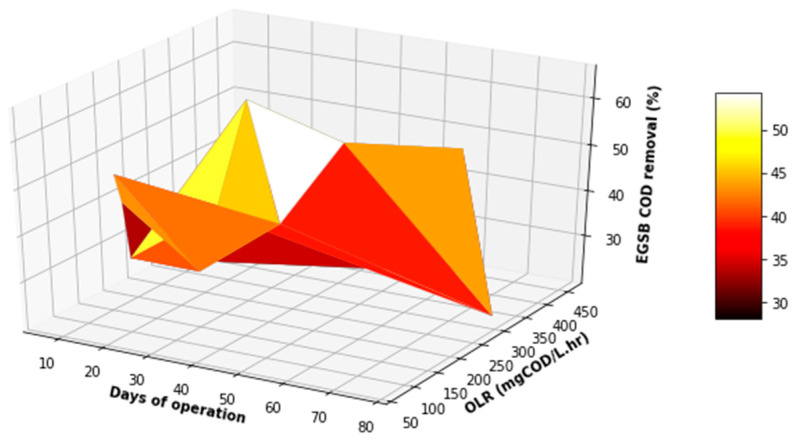
Variation in COD removal with differentiated OLR during the EGSB operational time.

**Figure 13:**
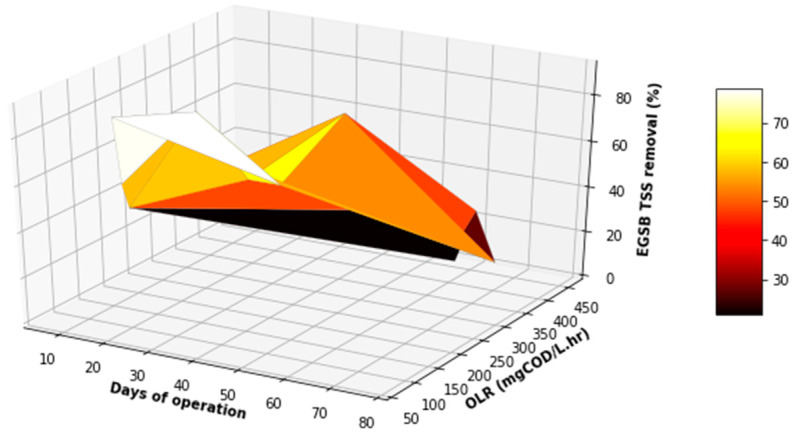
Variation in TSS removal with differentiated OLR during the EGSB operational time.

Figure 11, Figure 12 and Figure 13 demonstrate some increase in the performance of the EGSB, albeit for sporadic periods during the study. This performance did not improve even with a varied organic loading rate. Overall, the EGSB performed best for the removal of the FOG and TSS with peak removal percentages above 80%, while the bioreactor performance was low for the removal of COD, with an average 60% removal. The sporadic underperformance of the EGSB was determined not to be related to the increase in the OLR, with the overall performance trend not displaying a depreciation in the removal percentages of key parameters analyzed with an appreciation of the OLR. This observation further highlights the importance of monitoring the primary treatment system closely, especially when anaerobic digestion is used as a key driver of the overall performance of the system designed.

### 3.3. Membrane Bioreactor (MBR) Performance

**Figure 14:**
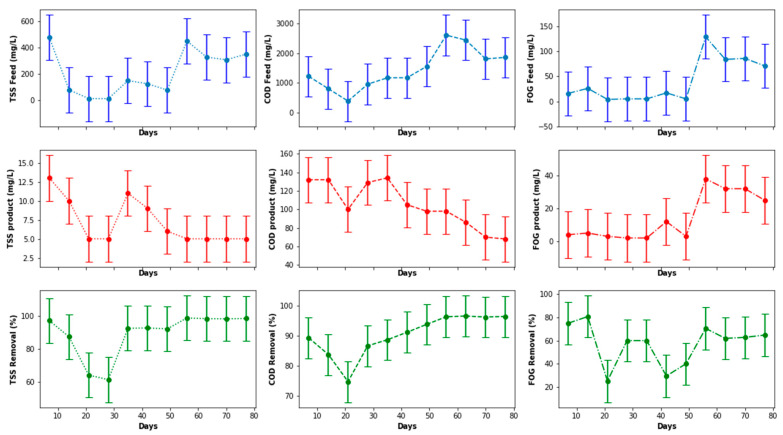
MBR performance with respect to COD, TSS, and FOG removal.

**Figure 15:**
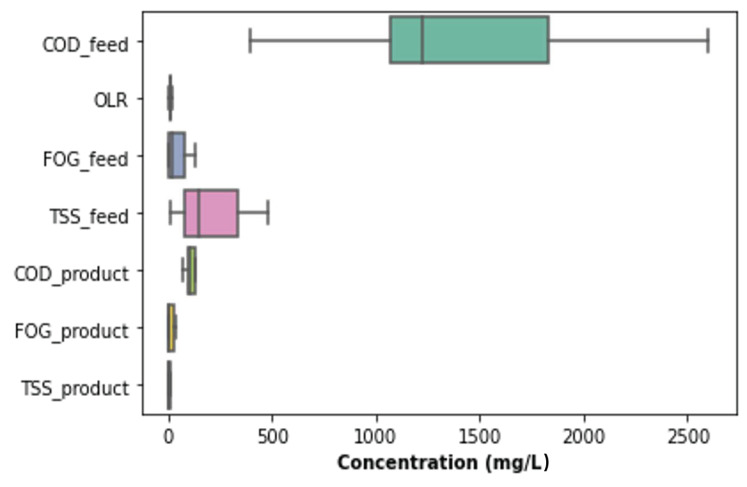
Boxplot of the MBR performance with regard to quantified quality parameters.

Figure 14 depicts the variation in FOG, TSS, and COD concentration in the feed and product streams of the MBR, including the percentage removal of these parameters. Due to a noticeable variation in the concentration of the parameters evaluated, an evaluation was carried out using the boxplot (Figure 15), which indicated that there was no outlier in each distribution. Despite a decrease in the TSS removal on the 21st and 28th days of operation, the performance of the MBR improved overtime with regard to COD and TSS removal. This trend was similar to that observed for the EGSB. The deterioration in the performance of the MBR on the 21st day of operation for the removal of the three parameters evaluated, as well as on the 28th day for TSS, was attributed to lower concentrations of contaminants in the feed to the MBR, which culminated in a lower performance because the feed was already of improved quality. However, this consistency in the performance of the MBR was not observed for FOG removal, which fluctuated between 20% and 80%, and it did not improve over time unlike that observed for TSS removal, which steadily remained above 60% with a peak at >95%. The COD removal was maintained above 75% throughout the process with a peak performance also being observed at >95%. This suggested that the structure of the membranes in the MBR was more suited to removing suspended solids and other nutrients than FOG, which was solubilized by the Ecoflush^TM^ used, suggesting seepage of solubilized FOG through the membranes. This assertion requires further investigation.

**Figure 16:**
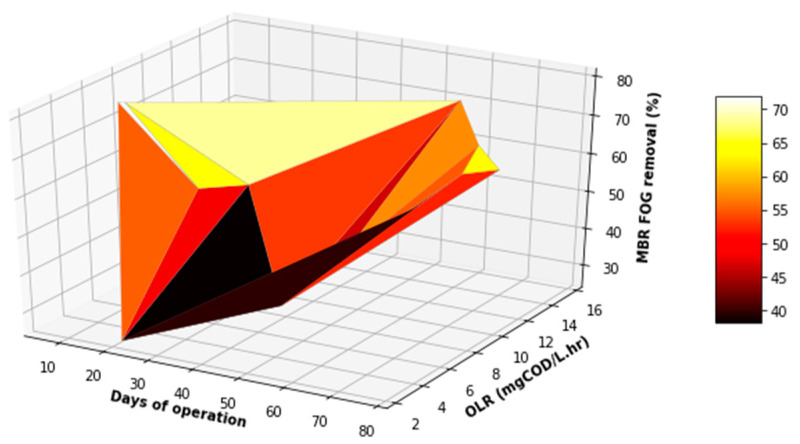
Variation in FOG removal with varying OLR during the operation of the MBR.

**Figure 17:**
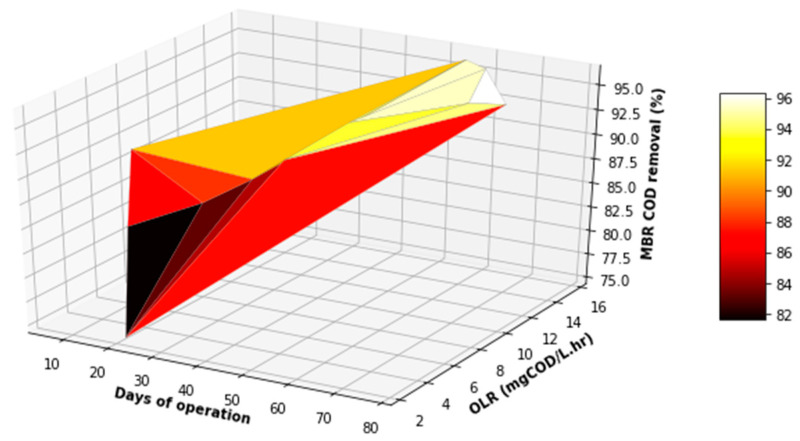
Variation in COD removal with varying OLR during the operation of the MBR.

**Figure 18:**
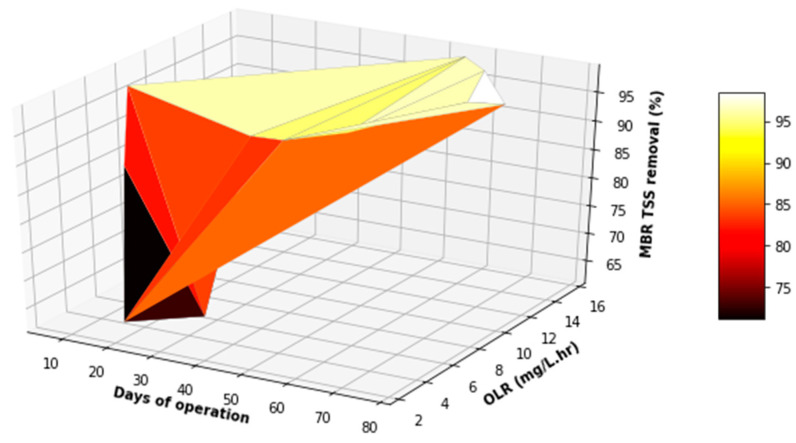
Variation in TSS removal with varying OLR during the operation of the MBR.

Figure 16, Figure 17 and Figure 18 provide a further evaluation of the performance of the MBR in terms of TSS, FOG, and COD removal with respect to the operating time and variation in the OLR to the system. It was observed that the range of the OLR was much less than that determined in the feed to the EGSB. This was attributed to a good performance of the EGSB that provided a feed with less organic matter to the MBR. These factors led to a more stable performance of the MBR system, even with fluctuation in the OLR throughout the experiment. The performance of the MBR was of significance and highly contributed to the overall performance of the lab-scale plant.

### 3.4. Overall System Performance of the Pretreatment–EGSB–MBR Lab-Scale System

**Figure 19:**
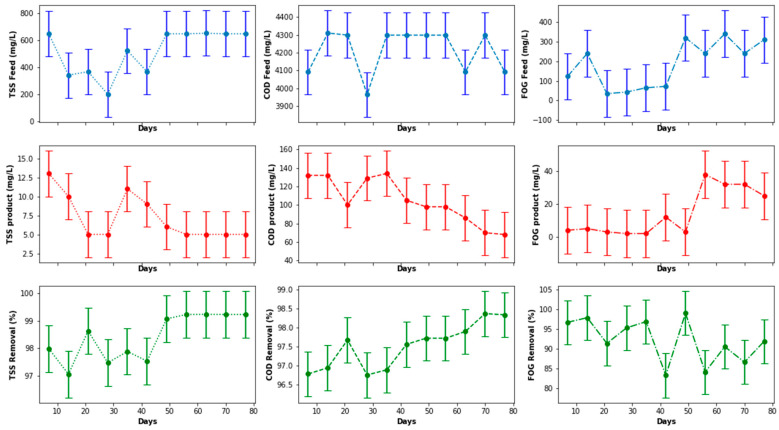
Overall performance of the pretreatment–EGSB–MBR system with respect to COD, TSS, and FOG removal.

**Figure 20:**
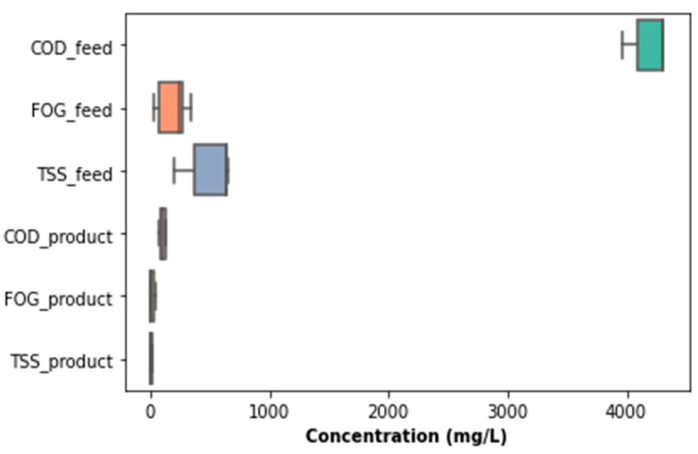
Boxplots of the parameters for the overall process.

**Figure 21:**
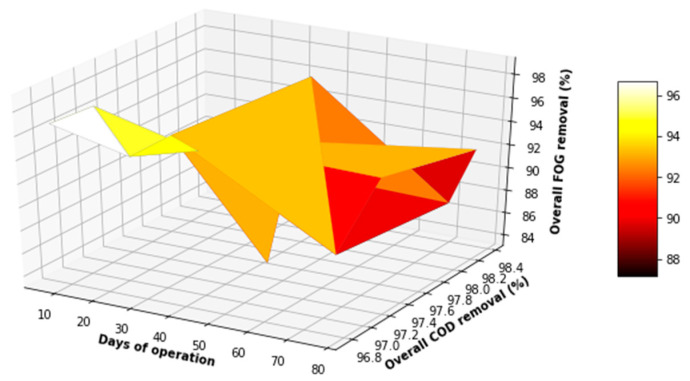
3D plot of the overall performance (COD VS FOG).

**Figure 22:**
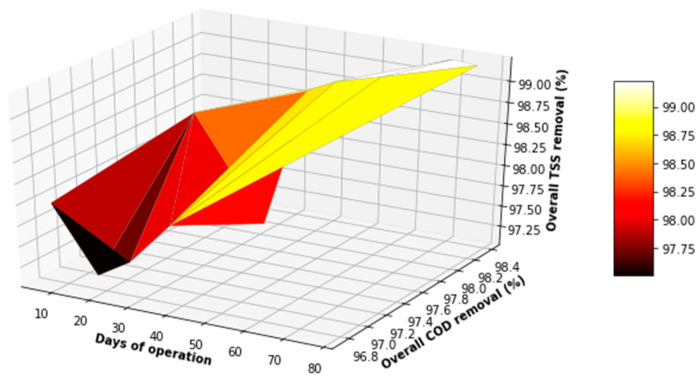
3D plot of the overall performance (COD VS TSS).

Figure 19 and Figure 20 showcases that there was an absence of outliers in the distribution of the parameters investigated for the overall process, which validated the assertion that the system was stable in its operation, with minimal variations in the key water quality parameters assessed for the overall process. From Figure 21 and Figure 22 below, it can be observed that the overall performance of the lab-scale plant varied between 97% and >99% for TSS removal, 96.5% and 99% for COD removal, and 84% and 98% for FOG removal. The overall performance of the lab-scale plant with respect to TSS and COD removal seemed more consistent when compared to FOG removal. There was demonstratable sporadic removal of the FOG percentage removal toward the end of the study; however, the overall system contributed to a significant decrease in the FOG concentration from the PSW with a concentration of less than 40 mg FOG/L in the final treated water, which is less than the limit of 400 mg FOG/L enforced by the CoCT for treated wastewater to be discharged to freshwater bodies.

Moreover, prior to replacing the outliers in the measured parameters for this study, a more representative distribution of key quality parameters was provided for the PSW samples collected for this study. The values of COD, FOG, and TSS concentration in the collected PSW samples all exceeded the discharge limits imposed by the CoCT by-laws (see Table 1). Such an excessive concentration in the wastewater quality parameters could adversely contaminate the environment, especially if the PSW is not treated. The quality of the PSW was also dependent on the prevailing operations in the poultry slaughterhouse from which the PSW samples were collected. Therefore, such wastewater should be treated to prevent harm to people and animals alike. No outliers being detected in the product from the EGSB or MBR of this study is indicative of the demonstrable robustness of the lab-scale system designed, despite the sporadic changes in PSW quality. The final wastewater output from the pretreatment–EGSB–MBR system met the discharge standards, and this finding can serve to promote such a technology for the treatment of medium- to high-strength wastewater, even in developing countries.

### 3.5. MBR Final Effluent Quality Compared to the Wastewater Discharge Standards

Table 1 provides a summary of the results obtained from the MBR outlet compared to wastewater discharge standards from several regulatory bodies, i.e., CoCT, Department of Water Affairs (DWA) 2010, and South African National Standards (SANS) 241:2015 for drinking water. It can be observed that parameters from the MBR outlet such as pH, temperature, conductivity, TDS, tCOD, TSS, and FOG were within the CoCT discharge standards, with only the conductivity not being within the DWA (2010) standards; a possible solution for conductivity might be to recommend continuous monitoring and maintenance of the poultry slaughterhouse wastewater pilot plant.

Furthermore, Table 2 lists the performance of similar technologies used in previous studies for the biological treatment of closely related wastewater, from which it was observed that the performance attained in this study was consistent with that observed using these technologies, although the operational conditions were not similar to those reported in previous studies. This is justified by overall peak COD, TSS, and FOG removal percentages above 98%, which is commendable given the fluctuations of the PSW fed to the system and the short period of acclimation used for the pretreatment–EGSB–MBR system.

## 4. Conclusions and Recommendations

A pretreatment–EGSB–MBR system was used to reduce the concentration of contaminants from a PSW. The pretreatment stage reached a peak performance of 50% for TSS removal, 80% for COD removal, and 82% for FOG removal. The EGSB also performed adequately with a peak removal percentage of 90% for TSS, >70% for COD, and >90% for FOG. Further removal was also observed using the MBR with the removal performance being >95% for both TSS and COD and 80% for FOG. These results culminated in a product with COD, TSS, and FOG concentrations being below the CoCT discharge standards. Moreover, the combination of a pretreatment unit with an EGSB and MBR demonstrated a robustness suitable for PSW treatment even with variations in OLR, highlighting the suitability of such a system for medium- to high-strength wastewater treatment for the poultry industry, which can be operated at low cost and with low energy requirements. It is recommended that a techno-economic analysis of the lab-scale design be undertaken to assess the feasibility of applying such a system on a larger scale in arid regions.

## Figures and Tables

**Table 1:** Results obtained from the MBR final effluent compared to standards.

Parameters	Units	MBR Outlet	CoCT Bylaws	DWA (2010)	SANS 241:2015
pH	n/a	7.7	5.5–9.5	5.5–9.5	5.5–9.5
Temperature	°C	22	≤40		
Conductivity	µs/cm	350	≤500	≤200	≤170
TDS	ppm	1000	4000		
tCOD	mg/L	110	≤5000	≤5000	1000–2400
TSS	mg/L	8	1000		
FOG	mg/L	27	400		

**Table 2:** Performance reached in similar wastewater treatment studies.

References	Technology Used	Type of Wastewater	Results
[12]	EGSB	PSW	69% tCOD removal; 98% TSS removal; 92% FOG removal
[12]	Ultrafiltration membrane bioreactor (UFMBR)	PSW	47% TSS removal; 62% tCOD removal
[12]	EGSB–UFMBR	PSW	92% tCOD removal; 99% TSS removal
[11]	EGSB	PSW	65% total COD removal
[23]	EGSB	Slaughterhouse wastewater	54–80% COD removal
[24]	MBR	Wastewater with high organic content	97% COD removal
[25]	Hollow fiber membrane filtration–EGSB	Domestic wastewater	85–96% COD removal
[13]	EGSB–MBR	Soft-drink industry wastewater	95% total COD removal
[14]	EGSB	Palm-oil mill effluent	91% tCOD removal
